# Mining Gene Expression Data for Pollutants (Dioxin, Toluene, Formaldehyde) and Low Dose of Gamma-Irradiation

**DOI:** 10.1371/journal.pone.0086051

**Published:** 2014-01-24

**Authors:** Alexey Moskalev, Mikhail Shaposhnikov, Anastasia Snezhkina, Valeria Kogan, Ekaterina Plyusnina, Darya Peregudova, Nataliya Melnikova, Leonid Uroshlev, Sergey Mylnikov, Alexey Dmitriev, Sergey Plusnin, Peter Fedichev, Anna Kudryavtseva

**Affiliations:** 1 Laboratory of Molecular Radiobiology and Gerontology, Institute of Biology of Komi Science Center of RAS, Syktyvkar, Russia; 2 Group of Postgenomic Studies, Engelhardt Institute of Molecular Biology of RAS, Moscow, Russia; 3 Ecological Department, Syktyvkar State University, Syktyvkar, Russia; 4 Laboratory of Genetics of Aging and Longevity, Moscow Institute of Physics and Technology, Dolgoprudny, Russia; 5 Department of Computational Systems Biology, Vavilov Institute of General Genetics, Moscow, Russia; 6 Department of Genetics, St. Petersburg State University, St. Petersburg, Russia; 7 Quantum Pharmaceuticals, Moscow, Russia; University of Texas Health Science Center at San Antonio/Greehey CCRI, United States of America

## Abstract

General and specific effects of molecular genetic responses to adverse environmental factors are not well understood. This study examines genome-wide gene expression profiles of *Drosophila melanogaster* in response to ionizing radiation, formaldehyde, toluene, and 2,3,7,8-tetrachlorodibenzo-p-dioxin. We performed RNA-seq analysis on 25,415 transcripts to measure the change in gene expression in males and females separately. An analysis of the genes unique to each treatment yielded a list of genes as a gene expression signature. In the case of radiation exposure, both sexes exhibited a reproducible increase in their expression of the transcription factors *sugarbabe* and *tramtrack*. The influence of dioxin up-regulated metabolic genes, such as *anachronism*, *CG16727,* and several genes with unknown function. Toluene activated a gene involved in the response to the toxins, *Cyp12d1-p*; the transcription factor *Fer3*’s gene; the metabolic genes *CG2065, CG30427,* and *CG34447*; and the genes *Spn28Da* and *Spn3*, which are responsible for reproduction and immunity. All significantly differentially expressed genes, including those shared among the stressors, can be divided into gene groups using Gene Ontology Biological Process identifiers. These gene groups are related to defense response, biological regulation, the cell cycle, metabolic process, and circadian rhythms. KEGG molecular pathway analysis revealed alteration of the Notch signaling pathway, TGF-beta signaling pathway, proteasome, basal transcription factors, nucleotide excision repair, Jak-STAT signaling pathway, circadian rhythm, Hippo signaling pathway, mTOR signaling pathway, ribosome, mismatch repair, RNA polymerase, mRNA surveillance pathway, Hedgehog signaling pathway, and DNA replication genes. Females and, to a lesser extent, males actively metabolize xenobiotics by the action of cytochrome P450 when under the influence of dioxin and toluene. Finally, in this work we obtained gene expression signatures pollutants (dioxin, toluene), low dose of gamma-irradiation and common molecular pathways for different kind of stressors.

## Introduction

Organisms are constantly exposed to the influence of adverse environmental factors. Among the most common adverse influences are formaldehyde, toluene, dioxins and low dose of ionizing radiation. Exposure to ionizing radiation in low doses (20 cGy or less) causes stochastic effects [1] and often leads to harmful long-term consequences in humans (e.g., leukemia) [2]. Formaldehyde is one of the most reactive pollutants, and has a wide range of household (wood-based panels, flooring materials, paper products, etc.) and industrial (as a feedstock for numerous industrial processes) sources [3], [4]. Acute and chronic exposure to formaldehyde has many health effects, such as allergies, neurotoxicity, pulmonary function damage, hematotoxicity, reproductive toxicity, genotoxicity, carcinogenesis, etc. [4]. Dioxins are persistent organic pollutants that are emitted from the incineration of solid waste and polyvinyl chloride, combustion of wood, automobile emissions, etc. [5] and accumulated in soil and water and are able to be accumulated in the human body [6]. They are the most potent synthetic poisons, being effective even at trace concentrations [5]. Toluene is an ingredient in organic solvents and dyes, and comes into contact with inhaled air. Toluene is an irritant capable of affecting the central nervous system and causing metabolic acidosis [7], [8]. Even at low concentrations, toluene causes oxidative stress and genotoxicity [9].

In recent years, dozens of papers have been published studying the expression of various genes under the action of dioxins [10], formaldehyde [11], [12], toluene [13], and ionizing radiation [14]. In this work, we compare the transcriptional signatures of four adverse factors in order to show exposure and sex-specific effects, based on our own experimental data. We investigate which cell response mechanisms induced by the pollutants are common to the different factors. This will expand our understanding of stress response mechanisms, which play an important role in the pathogenesis of many diseases and in aging processes. In high doses (dioxin, toluene and formaldehyde in our experiment), adverse effects (damage to DNA, proteins, and lipids) accumulate in the tissues in a deterministic manner that depends linearly on the dose. The mechanisms of the low-dose irradiation effects are the most obscure stress response mechanisms. In low doses the effects are stochastic, non-linear, and depend mainly on the efficiency of the stress response’s protective mechanisms, such as anti-radical protection, DNA repair, and detoxification of xenobiotics [15]. Any differences in the pattern of gene expression that depend on the influencing factors can be used to biosense each of the pollutants in low doses, providing a deeper understanding of their mechanisms of action and identifying both the similarities and differences in their action. For example, this sort of transcriptional signature identification has been performed for most drugs [16].

In this study, we use a systems approach to analyze the effects of formaldehyde, toluene, dioxin and low doses of ionizing gamma-irradiation on the expression of 25,415 transcripts obtained by RNA-seq analysis in adult wild-type (*Canton-S*) *Drosophila* males and females. Functional analysis identified exposure-specific genes and biological processes, and the overall effects reflect a non-specific stress response. We also observed that the sex-specific differences in the transcriptome are more pronounced than the effects on expression caused by the above-mentioned stressors.

## Materials and Methods

### *Drosophila Melanogaster* Strains

In our experiments, we used laboratory wild-type (*Canton-S*) males and females. The line was obtained from the collection at the Bloomington Drosophila Stock Center at Indiana University (Bloomington, USA).

### Flies Maintenance Conditions

The control- and experimental flies were maintained in the dark at T 25±0.5°C on a sugar-yeast medium containing 7 g of agar, 30 g of sugar, 8 g of dry yeast, 30 g of semolina, 4 ml of propionic acid, and 1 liter of water. Males and females were kept separately at densities of 20 flies of the same sex and age per 120 mL vial.

### Treatment by Ecotoxicants

For analyzes of the expression profiles the 5-day-old flies were used for each control- and experimental variants. For each variant 2 biological replicates were pooled. Number of flies for each control- and experimental variants in 2 biological replicates were 40.

Experimental flies were exposed to gamma-irradiation from ^226^Ra source with the dose rate of 36 mGy/h. The exposure time was 5 h 34 min; the absorbed dose –20 cGy. The dose of 20 cGy is the upper limit of low dose range of low-LET radiation [1]. The irradiation of *Drosophila* in doses of 40–60 cGy is known to induce the hormetic effect on the lifespan [17], [18].

In order to investigate the effect of 2,3,7,8-tetrachlorodibenzo-p-dioxin (TCDD) on gene expression profile, TCDD (Ekokhim, Russia), which was dissolved in toluene as solvent, was compounded with growth medium. Flies were incubated on growth medium with 0.822 µM/L TCDD for 3 days.

As toluene was a solvent for TCDD, we fed flies an equivalent amount (50 mM/L) of toluene (Sigma-Aldrich, USA) in growth medium as vehicle control for TCDD. Flies were incubated on growth medium with toluene for 3 days. To exclude specific influence of fresh media on gene expression it was changed in all control- and experimental variants simultaneously.

For treatment in the vapor of a 7% formaldehyde solution, flies were placed in a specially designed vial for 24 hours. The formaldehyde solution was produced using formalin (Panreac Química SLU, Spain) by diluting to the desired distillate concentration.

The concentrations and treatment conditions for the pollutants were adopted from the studies assessing the toxic effects of toluene [19] and formaldehyde [20] in *Drosophila*. The concentration of TCDD dependent of its content in toluene, and was about 20 times lower that TCDD concentration in *Drosophila* studies performed by other authors [21]. The used concentrations of pollutants demonstrated the low toxicity in *Drosophila*. However were much higher than maximum allowable concentrations (MAC). For example, according the Federal Drinking Water Standards the MAC for TCDD and toluene are 0.931×10^−7^ µM/L (3×10^−5^ µg/L) and 10.85 µM/L (1×10^3^ µg/L), respectively [22]. Thus the used concentrations of TCDD in 1×10^7^ and toluene – in 4.6×10^3^ times exceeded the MAC. According to the Occupational Safety and Health Administration, permissible exposure limits for occupational formaldehyde exposure are 0.75 ppm at or below an 8-hour time-weighted average and the short-term exposure limit of 2 ppm [23]. However we did not estimate the concentration of formaldehyde inside the vial.

The data from literature suggest that several hours of exposure is sufficient for the induction of changes in gene expression [24]. At the same time, the extra time after exposure can lead to the effects of unaccounted factors. The flies in the control- and experimental groups were fixed in liquid nitrogen immediately after treatment.

### RNA Isolation

Total RNA was extracted from 10 *Drosophila* images with ZR RNA MiniPrep™ (Zymo Research, USA) per the manufacturer’s instructions. The RNA quantity was determined using a Qubit® 2.0 Fluorometer (Invitrogen, USA) and the RNA integrity (RNA Integrity Score ≥8) was determined using an Agilent 2100 Bioanalyzer (Agilent, USA) per each manufacturer’s instructions.

### mRNA Library Preparation

To prepare samples for the mRNA sequencing libraries, we used the Illumina TruSeq™ RNA Sample Preparation Kit (Low-Throughput protocol) [25].

### Purification and Fragmentation of mRNA

In summary, 2.5–3.5 µg of total RNA from each sample of *Drosophila* was used to purify the poly-A containing mRNA molecules by poly-T oligo-attached magnetic beads, with two rounds of purification. During the second elution of poly-A RNA, the RNA was also fragmented and primed for cDNA synthesis according to the manufacturer’s protocol.

### cDNA Synthesis

The fragmented mRNA samples were subjected to cDNA synthesis according to the manufacturer’s protocol. Briefly, cDNA was synthesized from fragmented RNA using a SuperScript Double-Stranded cDNA Synthesis kit (Invitrogen, USA). The cDNA was then converted into double-stranded (ds) cDNA using the reagents supplied in the kit. Ampure XP beads were used to separate ds cDNA from the second-strand reaction mix.

### Preparation of cDNA Library

The double-stranded cDNA was subjected to library preparation using the Illumina TruSeq™ RNA sample preparation kit (Low-Throughput protocol) according to the manufacturer’s protocol. The ds cDNA was blunt-ended through an end-repair reaction. Next, a single ‘A’ nucleotide was added to the 3′ ends of the blunt fragments to prevent them from ligating to one another during the adapter ligation reaction. The multiple indexing adapters contain a single ‘T’ nucleotide on the 3′ end that provides a complementary overhang for ligating the adapter to the fragment. The cDNA fragments were then ligated to specific RNA Adapter Indexes supplied in the kit. The In-Line Control DNA was added to each enzymatic reaction. The controls contain ds DNA fragments designed to indicate the success or failure of specific enzymatic activity used in the library preparation process.

### DNA Fragment Enrichment

To selectively enrich DNA fragments with adapter molecules on both ends and to amplify the amount of DNA in the library, the PCR process was used according to the manufacturer’s protocol (15 cycles). All of the libraries were processed manually.

### Library Validation

The quantity of libraries was determined using a qPCR method on a AB 7500 Real-Time PCR System (Life Technologies, USA) according to the manufacturer’s protocol (Sequencing Library qPCR Quantification Guide) [25]. The primers matched sequences within adapters flanking an Illumina sequencing library. Before starting qPCR, a control template was selected to measure the libraries for quantification. The control template was a library with a known quantification (2 nM), template size (300 bp) and library type (transcriptome). The quality of libraries was determined on an Agilent 2100 Bioanalyzer (Agilent, USA) per the manufacturer’s instructions. The size and purity of the samples were checked. The final product was a band at approximately 260 bp.

### Transcriptome Sequence Assembly and Annotation

For sequencing and data analysis, cDNA libraries resulting from male *Drosophila* were combined in equal concentrations into a single pool and cDNA libraries resulting from female *Drosophila* were combined in equal concentrations into another pool. Each pool was sequenced in two lanes of the HiSeq™ 2000 sequencing platform (Illumina, Inc.) during the same sequencing run for a side-by-side comparison (100 bp single reads).

Image data output from the sequencing device were transformed into raw reads and stored in the FASTQ format. These data were filtered to remove raw reads that included the adapter sequence or which were of low quality. The transcriptome was assembled using Novoalign software and we used Berkeley Drosophila Genome Project (BDGP) assembly, release 5 (April 2006) as a reference. The number of reads per transcript was counted by the coverageBed application. Each of the libraries yielded approximately 50 million or more high-quality filtered sequences.

The data discussed in this publication have been deposited in NCBI’s Gene Expression Omnibus and are accessible through GEO Series accession number GSE50377 (http://www.ncbi.nlm.nih.gov/geo/query/acc.cgi?acc=GSE50377).

### Bioinformatics Analysis of Transcriptome Sequence Data

Most of the data analysis procedures were performed in the statistical programming environment R (version 3.1). We used the R package DESeq [26] to conduct comparisons of the genes for all experimental groups, using principal component analysis. PCA is the method of clustering of variance stabilized data to get sample-to-sample distances. It helps to overview over similarities and dissimilarities between samples. PCA was performed to group samples on the basis of their inter-varietal and inter-sex differences in the transcriptomes for 500 top (in order of decreasing of number of reads) most expressed genes in each sample. The values of the variables were standardized by subtracting their means and dividing by their standard deviation. Plot of the first two principal components (PCA1 and PCA2) is useful for visualizing the overall effect of experimental covariates.

We identified differentially expressed genes by comparing all treated groups with an intact control in the R package DSS [27] at False Discovery Rate control (FDR) <0.05 [28]. Significant gene lists (with p-values, FDRs and log fold changes) are attached as an Excel Table S1. We analyzed the overlap of lists of differentially expressed genes for different exotoxicants using the R package VennDiagram [29]. The list of overlapped genes and their functions is presented in Table S2 (genes common to the three or more impacts are in bold).

To analyze the functions of genes, we used “gene ontology” (GO) devoted to unifying the attributes of genes and gene products of all species [30]. A GO identifier was obtained using the R package biomaRt [31], [32]. We analyzed and compared impacts using biological process (BP-GO) annotations in the R package GeneAnswers, based on the Hypergeometric test [33].

In addition to the BP-GO analysis, comparisons were made using KEGG analysis for different exposures. KEGG is a source of annotations of molecular pathways for particular genes, provided by the Kyoto Encyclopedia of Genes and Genomes (www.genome.jp/kegg). For KEGG analysis, we selected the top 100 differently expressed genes (in order of increasing of false discovery rate, FDR<0.05) in flies under the four different stressors in comparison with the control. We divided top-lists of differentially expressed genes into pathways, using KEGG Mapper tool (http://www.kegg.jp/kegg/tool/map_pathway1.html). (Table S3). The table shows the number of genes included in each of the pathways and lists of these genes before enrichment. Each top-list was clustered based on the distance between genes and then enriched with the other fly genes included in the clusters. The distance between genes was calculated using the string-db.org database. The new list of genes was clustered using KEGG-mapper and finally a list of pathways was obtained. Each pathway was assigned an enrichment-score, which was calculated as the ratio of the number of genes from the list included in the pathway to the number of genes in every pathway. We consider a score reliable if it was not less than 0.5.

### qPCR Verification of RNA-Seq Results

qPCR was performed with the primers and probes listed in Table 1 using a 7500 Real-Time PCR System (Applied Biosystems, USA) by the following program: 95°C, 10 min; 40 cycles of 95°C, 10 s; 60°C, 60 s. Each reaction was repeated three times. The nucleotide sequences of the amplicons were verified by sequencing in 3730 DNA Analyzer automated sequencer (Applied Biosystems, USA). QPCR data were analyzed using four reference genes *RpL32*, *Actin*, *EF1alpha* [34] and *betaTub* [35] and the relative quantification or ΔΔCt-method [36]. Relative mRNA level was calculated by the following formula:
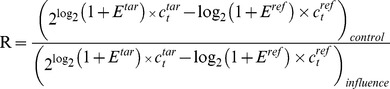
where *E* – efficiency of reaction, Ct – threshold cycle, ref – reference gene, tar – target gene. All efficiencies were more than 90%. All calculations performed using our program ATG (Analysis of Transcription of Genes) [37], [38], compatible with Relative Quantification (RQ) software (Applied Biosystems, USA). At least 1.5-fold mRNA level changes were considered as significant because of reference genes mRNA level variability.

**Table 1:** Primers and probes for qPCR analysis.

Gene	Primers	Probe*
*sug*	F: CAAGACCTTCAAGCACAGCA	20
	R: GTACGCTGGGCAGAGTGAG	
*ttk*	F: CGCGCTGAGTTGTGAAAAT	38
	R: CGCAGTTGTTAAACCGAAATTA	
*ana*	F: ACTTGCGACCAGGCAGAC	56
	R: CTTAAACGGTTCTGTTCGGTAAA	
*CG16727*	F: CCGAAACGCTTCACAAAAA	27
	R: CTCATGCTCGGTCAGACTTTC	
*Cyp12d1-p*	F: AAATCAGGATGCACTGGAAAA	67
	R: CTCATAATCACCGCCACCTT	
*Fer3*	F: CCGGATGTTCCATTCCAG	70
	R: GAGCGCTGCCACAGAGAC	
*CG2065*	F: GCTGCTGGACGTATTGAAGA	64
	R: GTTTTAATGAAACCTTGGGTGTG	
*CG34447*	F: CGACTGTTTGGCTACCTTTGT	25
	R: CAATATCGGGTTGGCACTG	

Note: F – forward primer; R – reverse primer; * – probe number from UPL (Universal Probe Library, Roche, Switzerland).

## Results

**Figure 1:**
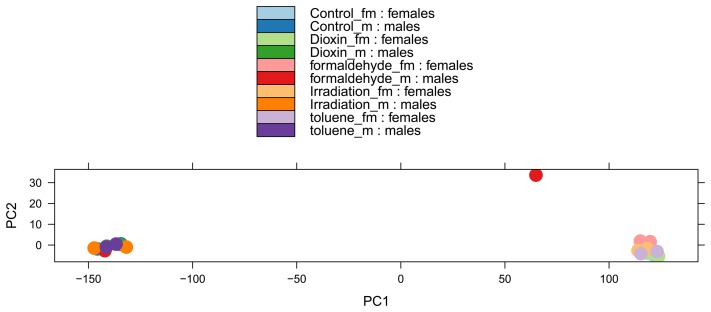
Principal components biplot on variance stabilized data, color-coded by condition-sex. PCA scores plot obtained from analysis of gene expression profiles. Proportion of the variance explained is 97.1% for PC1 and 1.1% for PC2.

Principal Component Analysis (PCA) algorithm reduces the dimensionality of such complex data as gene expression retaining most of the data set variation. Such reduction is achieved by identifying directions of the maximal variation between data. These directions - principal components - are further used for studied samples comparative analysis. The first principal component is the direction of the largest variation between samples. The variation, which is not correlated with this component, is used for revealing the next level principal component. The important feature of each gene expression dataset is the number of principal components explaining the sample variation. A comparison of the expression of the genes, based on principal component analysis, showed good agreement between the data for both sexes and replicates, except for one of the groups of formaldehyde exposure in males. Interestingly, the effects of stressors on transcriptome relative to the control were less pronounced than the differences between males (left part of Figure 1) and females (right part of Figure 1).

The significantly differentially expressed transcripts were as follows. In males after irradiation, 122 were up-regulated and 258 were down-regulated. In females after irradiation, 410 were up-regulated and 323 were down-regulated. In males after dioxin, 289 were up-regulated and 442 were down-regulated. In females after dioxin, 1038 were up-regulated and 441 were down-regulated. In males after toluene, 257 were up-regulated and 737 were down-regulated. In females after toluene, 1003 were up-regulated and 1188 were down-regulated. In males after formaldehyde, 6 were up-regulated and 51 were down-regulated. In females after formaldehyde, 4 were up-regulated and 8 were down-regulated. The largest number of genes was altered in both males and females by the action of dioxin and toluene. The smallest number of genes was altered by the action of formaldehyde.

**Figure 2:**
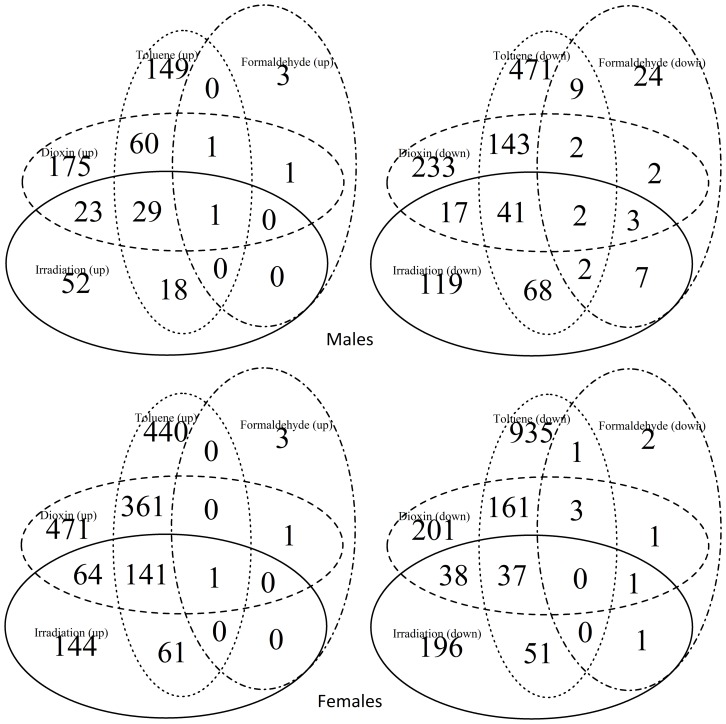
Diagrams representing the quantity of shared genes between the different treatments.

A comparison of lists of differentially expressed genes for different effects obtained with an FDR <0.05 showed that, in both males and females, the action of toluene and dioxin produced the most similar change in genes (Figure 2). On the one hand, this is because it was under these influences the highest quantity of genes altered their expression. On the other hand, the use of a small amount of toluene as a solvent for dioxin causes a cross effect on both factors. The next most similar sets of changes were for ‘dioxin and radiation’ and ‘toluene and radiation’. But there are also a lot of genes common not for two but for three and four treatments (Table S2, highlighted in bold).

**Figure 3:**
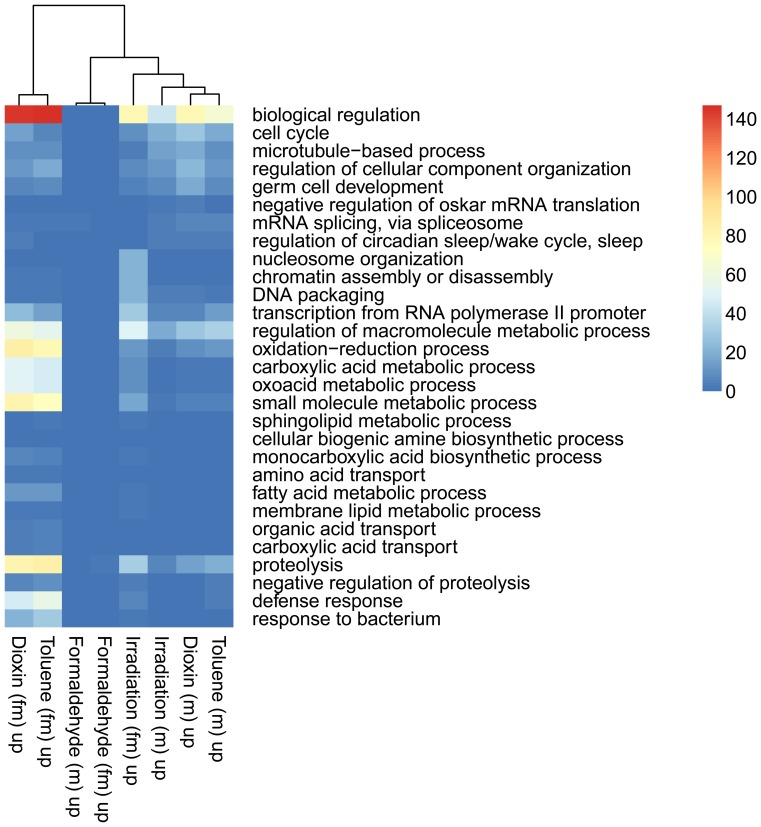
Heat map of up-regulated biological processes based on Gene Ontology IDs for treated males (m) and females (fm). The color scale indicates the number of genes in a group.

**Figure 4:**
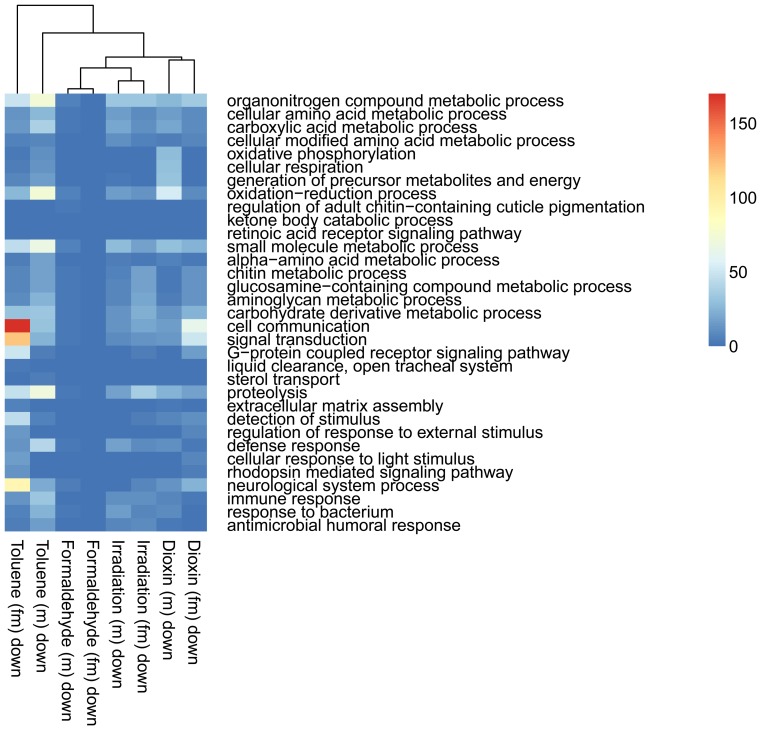
Heat map of down-regulated biological processes based on Gene Ontology IDs for treated males (m) and females (fm). The color scale indicates the number of genes in a group.

**Figure 5:**
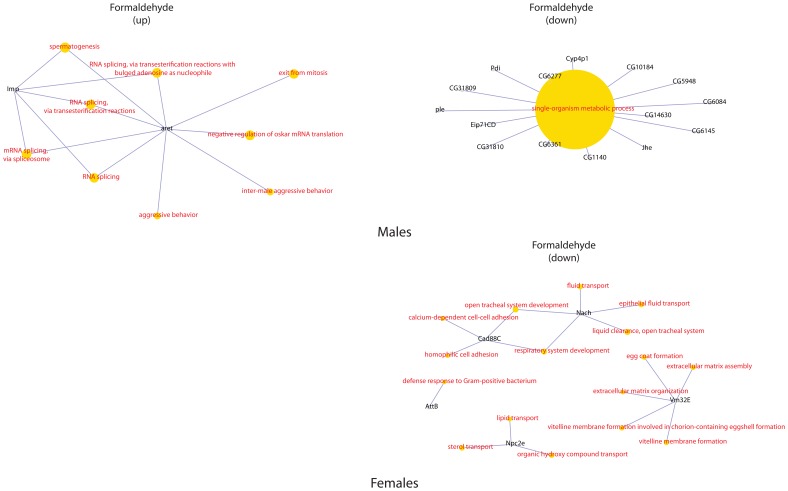
Gene-concept networks by gene ontology analysis for formaldehyde treated males and females. Figures 5–8 represent gene-concept networks by gene ontology analysis using the Bioconductor GeneAnswers Package. Yellow nodes are gene ontology terms, gray nodes correspond to differential expressed genes from the RNA-Seq data. The sizes of the centroid nodes reflect p-values of the gene associations as calculated by GeneAnswers. *Genes, up-regulated in males:* Upregulated genes were characterized by their importance for such functions as RNA splicing, aggressive behavior, spermatogenesis, exit from mitosis. *Genes, down-regulated in males:* All genes, enriched with unknown and those with low quality annotation, were related mostly to single-organism metabolic process functional category. *Genes, down-regulated in females:* Only a few multifunctional genes were characterized with significant downregulation, including Vm32E, involved in extracellular matrix organization and assembly, Npc2c, important for sterol and lipid transport, Cad88C and Nach, important for fluid transport and cell adhesion. The AttB gene, involved in defense response to Gram-positive bacterium, was also downregulated.

**Figure 6:**
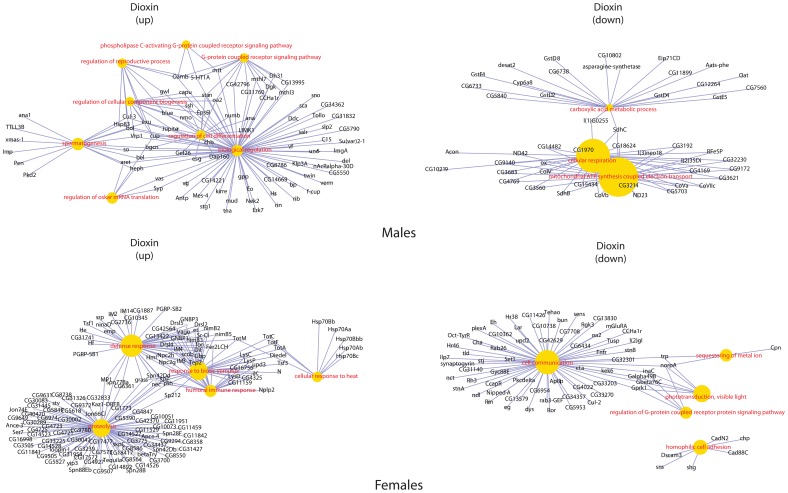
Gene-concept networks by gene ontology analysis for dioxin treated males and females. *Genes, up-regulated in males:* Most of upregulated gene the involved in biological regulation, G-protein coupled receptor signaling pathway and regulation of cell differentiation. Smaller functional groups of genes, involved in the regulation of oskar mRNA translation, spermatogenesis, regulation of reproductive process and phospholipase C-activating G-protein coupled receptor signaling pathway were also upregulated. *Genes, down-regulated in males:* The genes characterized with decreased expression were mostly known to be involved in the cellular respiration and related functional category of mitochondrial ATP synthesis coupled electron transport. Small independent cluster of carboxylic acid metabolic process was also downregulated. *Genes, up-regulated in females:* Most of genes were annotated as involved in the processes of the proteolysis, defense response, and response to biotic stimulus. The smaller clusters of cellular response to heat and humoral immune response were revealed. *Genes, down-regulated in females:* The most of downregulated genes were annotated as involved in cell communication. Related functional categories, such as homophilic cell adhesion, were also revealed.

**Figure 7:**
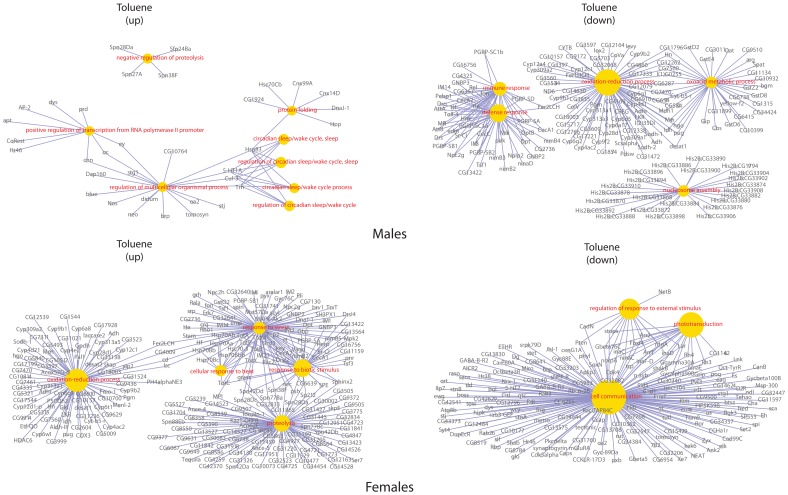
Gene-concept networks by gene ontology analysis for toluene treated males and females. *Genes, up-regulated in males:* Upregulated genes were known to be involved in the protein folding, circadian sleep/wake regulation, positive regulation of transcription from RNA polymerase II promoter and proteolysis regulation. *Genes, down-regulated in males:* The large number of downregulated genes in this treatment were functionally clustered to four main groups: response to stress (including related functional category of response to heat), response to biotic stimulus, proteolysis and oxidation-reduction process. *Genes, up-regulated in females:* The overexpressed genes were functionally clustered into four main clusters: oxidation-reduction process, proteolysis, response to stress, response to biotic stimulus and the smaller cluster of cellular response to heat. *Genes, down-regulated in females:* The cell communication functional category was extremely downregulated in this treatment. Smaller gene clusters, involved in the phototransduction and regulation of response to external stimulus, were also revealed.

**Figure 8:**
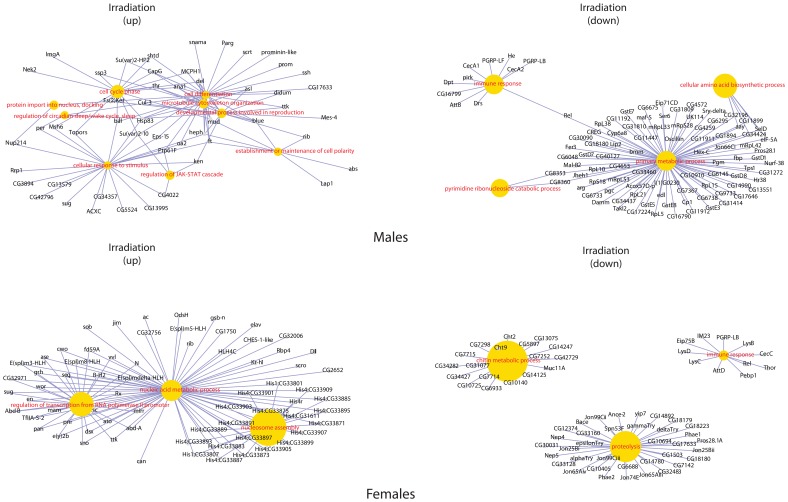
Gene-concept networks by gene ontology analysis for radiation treated males and females. *Genes, up-regulated in males:* The most representative functional groups of genes were annotated as involved in the cell differentiaion, develpmnetal process involved in reproduction, cell cycle phase and cellular response to stimulus. The JAK-STAT cascade regulators were also revealed. *Genes, down-regulated in males:* Mostly downregulated genes were known to be involved in primary metabolism, including amino acid biosynthesis. The smaller cluster of immune response genes was also downregulated. *Genes, up-regulated in females:* Upregulated genes were clustered into three main functional groups: genes, annotated as involved into nucleic acid metabolic process, nucleosome assembly and regulation of transcription from RNA polymerase II promoter. *Genes, down-regulated in females:* The main functional gene groups, downregulated in this set, were known to be involved in proteolysis, immune response and chitin metabolism.

Clustering the biological processes controlled by up-regulated genes showed high convergence of data between males and females for each influence (Figure 3). Clustering repressed genes processes (Figure 4) revealed a convergence of the effects of exposure, for both sexes. Formaldehyde caused the smallest effects in biological processes – such processes as reproductive activity, splicing, aggressive behavior and regulation of cell cycle were up-regulated only in males. Cellular activity, cellular transport, intracellular signaling, reproductive activity, and the process of development of respiratory system were decreased in females. And in males the activity of genes responsible only for metabolism was down-regulated (Figure 5). In females, dioxin and toluene led to an increased defense response (especially to bacterial contamination), oxidation-reduction processes, and proteolysis, but activity of such function as cell communication, phototranspiration and response to stimulus (only after the exposure of toluene is decreased) (Figure 6, 7). In males, toluene induced up-regulation in the metabolic control of macromolecules and circadian rhythms and down-regulation in different types of oxidation-reduction, immune response and nucleosome assembly (Figure 7). Dioxin led to an increasing of reproductive processes, intracellular signaling pathway, process of cell differentiation and transcription and decreasing of cellular respiration, carboxylic and metabolic processes (Figure 6). The influence of radiation on the females led to the activation of genes, induced by chromatin rearrangements and the genes of metabolism of macromolecules and deactivation of proteolysis, immune response and chitin metabolic process. In males radiation caused such effects as promoting different cellular processes, circadian rhythms, regulation of JAK-STAT cascade, developmental process in reproduction, and deactivation of immune response, metabolic and cellular processes (Figure 8).

For each of the studied factors the most significant changes up-regulated processes are regulation of the cell cycle, formation of gametes, circadian rhythms, splicing, proteolysis, and various aspects of metabolism. Repressed genes are responsible for cell respiration, cell-cell communication, and various aspects of metabolism, immune response, and response to light stimuli. The processes of cell-cell signaling and signal transduction were most depressed in females after exposure to toluene and dioxin.

**Figure 9:**
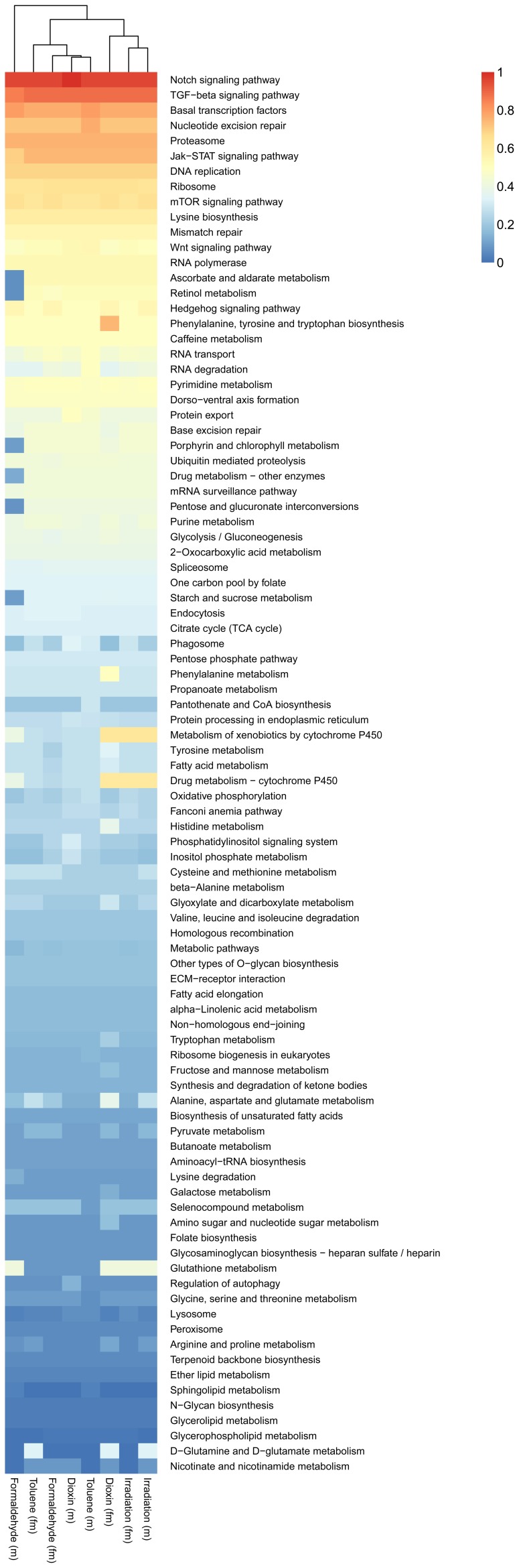
Heat map of molecular pathways based on KEGG analysis IDs for treated males (m) and females (fm), color-coded by enrichment-score.

An analysis of the molecular pathways by KEGG showed a significant change in the processes of stress response, cell-cell signaling, and biosynthetic pathways (Figure 9).

Remarkably, the transcriptome changes against all the studied types of stresses are similar; they involve differential regulation of a large common cluster of the genes, most of them earlier identified as related to genome maintenance or aging.

Ascorbate and aldarate metabolism and retinol metabolic pathways are evident in the lists for dioxin, formaldehyde, irradiation, and toluene. The Notch signaling pathway, TFG-beta signaling pathway, proteasome, basal transcription factors, nucleotide excision repair, Jak-STAT signaling pathway, circadian rhythm, Hippo signaling pathway, mTOR signaling pathway, ribosome, mismatch repair, RNA polymerase, mRNA surveillance pathway, Hedgehog signaling pathway, caffeine metabolism, and DNA replication pathways are evident in the lists for all pathways. Interestingly, one of predicted longevity pathways of caffeine metabolism consists of only two genes and has a score of 0.5 in every pathway list.

We also have done mining of unique to each treatment genes and have revealed several such genes (Table 2), up- and down-regulated especially for toluene, dioxin, formaldehyde or low doze irradiation. The sequencing data were validated using qPCR for unique to each treatment genes with statistically valid expression level increase in both males and females. Relative mRNA level (R) of 8 genes under different influences is represented in Table 3. The increase of expression level (up to 2.6-fold) was observed in all tested cases except one (*ttk* gene under ionizing radiation influence in male). Obtained qPCR data were concordant with the sequencing data.

**Table 2:** List of unique genes which are up- and down-regulated in both sexes after a particular treatment.

Gene symbol	Gene name*	Molecular and biological function	References	Gene Ontology Term***	InterPro Term***
**Up-regulated genes**					
**Irradiation**					
*sug*	*sugarbabe*	Transcription factor which represses a set of lipase genes involved in fat catabolism	[60]	transport, determination of adult lifespan, biological_process, aging, cellular_component, integral to membrane, transmembrane transport	Major facilitator superfamily domain, general substrate transporter, Major facilitator superfamily, Major facilitator superfamily associated domain
*ttk*	*tramtrack*	Transcription factor with repressor activity, expressed downstream of Notch	[63]–[65]	inter-male aggressive behavior, regulation of cell shape, transcriptional repressor complex, DNA binding, imaginal disc-derived wing morphogenesis, negative regulation of transcription, DNA-dependent, regulation of tube size, open tracheal system, protein binding, dendrite morphogenesis, intracellular, cell fate determination, protein complex, locomotion, tracheal outgrowth, open tracheal system, dorsal trunk growth, open tracheal system, R7 cell development, cell morphogenesis, neurological system process, cell differentiation, biological_process, ovarian follicle cell development, molecular_function, regulation of embryonic cell shape, growth, positive regulation of transcription from RNA polymerase II promoter, nucleus, compound eye cone cell differentiation, polytene chromosome, neuron development, locomotion involved in locomotory behavior, cellular_component, chromatin binding, protein homodimerization activity, chromosome, nucleoplasm, negative regulation of transcription from RNA polymerase II promoter, chitin-based cuticle development, regulation of transcription from RNA polymerase II promoter, dorsal appendage formation, brain morphogenesis, startle response, branch fusion, open tracheal system, peripheral nervous system development, zinc ion binding, reproduction, anatomical structure development, anatomical structure formation involved in morphogenesis, ion binding, cell, sequence-specific DNA binding transcription factor activity, branching involved in open tracheal system development, RNA polymerase II core promoter proximal region sequence-specific DNA binding, cellular component assembly, RNA polymerase II core promoter proximal region sequence-specific DNA binding transcription factor activity involved in negative regulation of transcription, nucleic acid binding transcription factor activity, RNA polymerase II distal enhancer sequence-specific DNA binding, compound eye corneal lens morphogenesis, R1/R6 development, organelle	BTB/POZ-like, BTB/POZ, Zinc finger, C2H2, AT hook, DNA-binding motif, BTB/POZ fold, Zinc finger, C2H2-like, Zinc finger C2H2-type/integrase DNA-binding domain
**Dioxin**					
*ana*	*Anachronism*	Secreted glial glycoprotein; inhibits neuroblast proliferation, is homologous to Transforming Growth Factor-beta, N-terminal	[66], [67]	growth factor activity, extracellular region, cell differentiation, biological_process, molecular_function, negative regulation of neuroblast proliferation, growth, cellular_component, anatomical structure development	Transforming growth factor-beta, N-terminal
*CG16727*	*CG16727*	Organic cation transmembrane transporter	[68]	transport, organic cation transmembrane transporter activity, biological_process, molecular_function, cellular_component, integral to membrane, transmembrane transport, transmembrane transporter activity	Major facilitator superfamily domain, Major facilitator superfamily domain, general substrate transporter, General substrate transporter
*nimC2*	*Nimrod C2*	Encode proteins containing various number of NIM repeats, phagocytosis,	[69]	transport, protein binding, biological_process, vesicle-mediated transport, phagocytosis, molecular_function, cellular_component, integral to membrane	Cysteine rich repeat, tripleX, Epidermal growth factor-like domain
*CG13116*	*CG13116*	Unknown function	**	No data	No data
*CG14082*	*CG14082*	Unknown function	**	No data	No data
*CG13075*	*CG13075*	Unknown function	**	extracellular region, biological_process, molecular_function, chitin binding, cellular_component, chitin metabolic process	No data
*CG34386*	*CG34386*	Unknown function	**	No data	No data
**Toluene**					
*Cyp12d1-p*	*Cyp12d1-p*	Cytochrome P450, E-class, group I; response to insecticide	[70]–[72]	oxidoreductase activity, iron ion binding, cytoplasm, response to insecticide, intracellular, response to DDT, heme binding, biological_process, molecular_function, electron carrier activity, cellular_component, mitochondrion, oxidoreductase activity, acting on paired donors, with incorporation or reduction of molecular oxygen, ion binding, cell, oxidation-reduction process, organelle	Cytochrome P450, Cytochrome P450, conserved site, Cytochrome P450, E-class, group I
*Fer3*	*Fer3*	Transcription factor that binds to the E-box and functions as inhibitor of transcription. DNA binding requires dimerization with an E protein. Inhibits transcription activation by ASCL1/MASH1 by sequestering E proteins	[73], [74]	regulation of transcription, DNA-dependent, intracellular, biological_process, molecular_function, protein dimerization activity, nucleus, cellular_component, cell, sequence-specific DNA binding transcription factor activity, nucleic acid binding transcription factor activity, organelle	Helix-loop-helix domain
*CG2065*	*NADP-retinol dehydroge*	Oxidoreductase; takes part in the metabolic process	[75], [76]	oxidoreductase activity, metabolic process, biological_process, molecular_function, nucleotide binding	Glucose/ribitol dehydrogenase, Short-chain dehydrogenase/reductase SDR, NAD(P)-binding domain
*CG30427*	*CG30427*	Fatty acyl-CoA reductase; involved in phagocytosis and determination of adult lifespan	[77]	oxidoreductase activity, transport, phagocytosis, engulfment, biological_process, vesicle-mediated transport, molecular_function, nucleotide binding, fatty-acyl-CoA reductase (alcohol-forming) activity, membrane organization	Male sterility, NAD-binding, NAD(P)-binding domain, Fatty acyl-CoA reductase
*CG34447*	*CG34447*	Triglyceride lipase, involved in lipid metabolic process	**	triglyceride lipase activity, biological_process, molecular_function, lipid metabolic process, cellular_component	Lipase, Lipase, N-terminal
*Spn28Da*	*Serpin 28Da*	Serine-type endopeptidase inhibitor; involved in reproduction	[78], [79]	serine-type endopeptidase inhibitor activity, negative regulation of proteolysis, enzyme regulator activity, biological_process, molecular_function	Serpin domain, Protease inhibitor I4, serpin, conserved site, Serpin family
*Spn3*	*Serpin 38F*	Serine-type endopeptidase inhibitor, involved in reproduction and defense response to Gram-negative bacterium	[80]–[85]	serine-type endopeptidase inhibitor activity, negative regulation of proteolysis, extracellular space, response to stress, extracellular region, enzyme regulator activity, biological_process, molecular_function, defense response to Gram-negative bacterium, cellular_component, reproduction, multicellular organism reproduction	Serpin domain, Serpin family
*CG13131*	*CG13131*	Unknown function	**	No data	No data
**Down-regulated genes**					
**Irradiation**					
*CG11891*	*CG11891*	Unknown function	**	molecular_function, transferase activity, transferring phosphorus-containing groups	No data
*CG13323*	*CG13323*	Unknown function	**	No data	No data
*CG18180*	*CG18180*	Serine-type endopeptidase activity, proteolysis with a possible role in immune function	[86]	peptidase activity, biological_process, molecular_function, proteolysis, catalytic activity, serine-type endopeptidase activity	Peptidase cysteine/serine, trypsin-like, Peptidase S1/S6, chymotrypsin/Hap, active site, Peptidase S1A, chymotrypsin-type, Peptidase S1/S6, chymotrypsin/Hap
*CG42751*	*CG42751*	Unknown function	**	No data	No data
*CG6295*	*CG6295*	Hydrolase, lipid metabolic process	[87]	triglyceride lipase activity, biological_process, molecular_function, lipid metabolic process, phosphatidylcholine 1-acylhydrolase activity	Lipase, Lipase, N-terminal, Dol/Ves 1 allergen
*CG6675*	*CG6675*	Hydrolase, lipid metabolic process	**	lipase activity, biological_process, molecular_function, lipid metabolic process	Lipase, Lipase, N-terminal
*CG9360*	*CG9360*	Oxidoreductase activity	**	oxidoreductase activity, oxidoreductase activity, acting on CH-OH group of donors, metabolic process, biological_process, molecular_function, nucleotide binding	Glucose/ribitol dehydrogenase, Short-chain dehydrogenase/reductase SDR, NAD(P)-binding domain
*Cyp6a2*	*Cytochrome P450–6a2*	Is involved in the breakdown of synthetic insecticides and involved in the metabolism of insect hormones.	[88]	oxidoreductase activity, iron ion binding, response to insecticide, intracellular, membrane, heme binding, intracellular membrane-bounded organelle, biological_process, molecular_function, response to caffeine, electron carrier activity, cellular_component, oxidoreductase activity, acting on paired donors, with incorporation or reduction of molecular oxygen, ion binding, cell, oxidation-reduction process, organelle	Cytochrome P450, Cytochrome P450, conserved site, Cytochrome P450, E-class, group I
*Cyp6a20*	*Cyp6a20*	Monooxygenase, oxidoreductase, electron carrier activity, heme binding, iron ion binding, takes part in aggressive behavior and defense response to Gram-negative bacterium	[89]	oxidoreductase activity, iron ion binding, intracellular, membrane, aggressive behavior, heme binding, intracellular membrane-bounded organelle, biological_process, molecular_function, electron carrier activity, cellular_component, oxidoreductase activity, acting on paired donors, with incorporation or reduction of molecular oxygen, ion binding, cell, oxidation-reduction process, organelle	Cytochrome P450, Cytochrome P450, conserved site, Cytochrome P450, E-class, group I
*Fer3*	*Fer3*	Transcription factor that binds to the E-box and functions as inhibitor of transcription. DNA binding requires dimerization with an E protein. Inhibits transcription activation by ASCL1/MASH1 by sequestering E proteins	[73], [74]	regulation of transcription, DNA-dependent, intracellular, biological_process, molecular_function, protein dimerization activity, nucleus, cellular_component, cell, sequence-specific DNA binding transcription factor activity, nucleic acid binding transcription factor activity, organelle	Helix-loop-helix domain
*GstE3*	*Glutathione S transferase E3*	Glutathione transferase activity, response to oxidative stress, resistance to insecticides	[90], [91]	protein binding, glutathione transferase activity, cellular amino acid metabolic process, transferase activity, transferring alkyl or aryl (other than methyl) groups, biological_process, molecular_function, glutathione metabolic process, sulfur compound metabolic process, small molecule metabolic process	Glutathione S-transferase, C-terminal, Glutathione S-transferase, N-terminal, Glutathione S-transferase, C-terminal-like, Thioredoxin-like fold, Glutathione S-transferase/chloride channel, C-terminal
*Keap1*	*Keap1*	Actin binding, defends organisms against the detrimental effects of oxidative stress	[92], [93]	response to oxidative stress, protein binding, response to stress, biological_process, molecular_function, cytoskeletal protein binding, actin binding	BTB/POZ-like, BTB/POZ, BTB/Kelch-associated, BTB/POZ fold, Kelch repeat type 1, Kelch-type beta propeller
**Dioxin**					
*CG12519*	*CG12519*	Unknown function	**	No data	No data
*CG18048*	*CG18048*	Methyltransferase, Transferase, tRNA processing	**	biological_process, molecular_function, methyltransferase activity, tRNA processing, tRNA metabolic process, cellular nitrogen compound metabolic process	Methyltransferase TRM13, TRM13/UPF0224 family, U11–48K-like CHHC zinc finger domain, Zinc finger, CCCH-type, TRM13
*HLH3B*	*Helix loop helix protein 3B*	Sequence specific DNA- binding proteins that act as transcription factors,control of cellular proliferation to tissue differentiation, takes part in neurogenesis	[94], [95]	intracellular, molecular_function, protein dimerization activity, nucleus, cellular_component, cell, sequence-specific DNA binding transcription factor activity, nucleic acid binding transcription factor activity, organelle	Helix-loop-helix domain
**Toluene**					
*Cda9*	*Chitin deacetylase-like 9*	Hydrolase activity, acting on carbon-nitrogen (but not peptide) bonds, catalyze the N-deacetylation of chitin to form chitosan	[96], [97]	hydrolase activity, acting on carbon-nitrogen (but not peptide) bonds, biological_process, molecular_function, carbohydrate metabolic process, catalytic activity	Glycoside hydrolase/deacetylase, beta/alpha-barrel, Polysaccharide deacetylase
*CG10357*	*CG10357*	The orthologous GABACl, takes part ininhibitory neurotransmission	[98]	triglyceride lipase activity, biological_process, molecular_function, lipid metabolic process	Lipase, Lipase, N-terminal
*CG14538*	*CG14538*	Unknown function	**	No data	No data
*CG15308*	*CG15308*	Unknown function	**	No data	No data
*CG15533*	*CG15533*	Sphingomyelin phosphodiesterase activity	[60]	catabolic process, biological_process, sphingomyelin phosphodiesterase activity, sphingomyelin catabolic process, molecular_function, lipid metabolic process, hydrolase activity, sphingomyelin metabolic process	Saposin B, Sphingomyelin phosphodiesterase, Metallophosphoesterase domain
*CG15534*	*CG15534*	Sphingomyelin phosphodiesterase activity	[60]	catabolic process, biological_process, sphingomyelin phosphodiesterase activity, sphingomyelin catabolic process, molecular_function, lipid metabolic process, hydrolase activity, sphingomyelin metabolic process	Saposin B, Sphingomyelin phosphodiesterase, Metallophosphoesterase domain
*CG34423*	*CG34423*	Negative regulation of catalytic activity, negative regulation of nucleotide metabolic process	**	cytoplasm, intracellular, negative regulation of nucleotide metabolic process, enzyme regulator activity, biological_process, molecular_function, cellular_component, enzyme inhibitor activity, mitochondrion, cell, organelle	ATPase inhibitor, IATP, mitochondria
*CG4452*	*CG4452*	Unknown function	**	No data	No data
*Cp7Fb*	*Chorion protein b at 7F*	Is essential for vitelline membrane integrity	[99]	chorion-containing eggshell formation, cell differentiation, biological_process, cellular_component, reproduction, chorion, anatomical structure development, anatomical structure formation involved in morphogenesis, cell, external encapsulating structure	No data
*Cpr72Ec*	*Cuticular protein 72Ec*	Structural constituent of cuticle	[100]	structural molecule activity, structural constituent of cuticle, molecular_function, structural constituent of chitin-based cuticle	Insect cuticle protein
*Dop2R*	*Dopamine 2-like receptor*	G-protein coupled receptor, function as autoreceptors and regulate the release of dopamine	[101], [102]	G-protein coupled receptor activity, signal transducer activity, biological_process, molecular_function, dopamine receptor signaling pathway, cellular_component, integral to membrane, signal transduction, G-protein coupled amine receptor activity, G-protein coupled receptor signaling pathway, dopamine neurotransmitter receptor activity, dopamine neurotransmitter receptor activity, coupled via Gi/Go	Dopamine receptor, GPCR, rhodopsin-like, 7TM, GPCR, rhodopsin-like
*Gr39a*	*Gustatory receptor 39a*	Chemoreception, gustatory response.	[103]–[105]	male courtship behavior, sensory perception of taste, neurological system process, signal transducer activity, biological_process, molecular_function, taste receptor activity, cellular_component, integral to membrane, reproduction	7TM chemoreceptor
*Npc1b*	*Niemann-Pick type C-1b*	Hedgehog receptor activity, takes part in molecular mechanisms by which dietary cholesterol is trafficked within cells	[106], [107]	dorsal closure, plasma membrane, embryo development, transport, membrane, signal transducer activity, biological_process, molecular_function, cellular_component, integral to membrane, hedgehog receptor activity, central nervous system development, peripheral nervous system development, intestinal cholesterol absorption, anatomical structure development, cell	Patched, Sterol-sensing domain
*Osi8*	*Osiris 8*	Integral to plasma membrane, is required for Toll activation by Gram-positive bacterial infection, takes part in the immune response,	[108], [109]	plasma membrane, biological_process, molecular_function, integral to plasma membrane, cellular_component, cell	Protein of unknown function DUF1676
*RpL7-like*	*Ribosomal protein L7-like*	Ribonucleoprotein, ribosomal protein, takes part in neurogenesis	[110]	cytosol, ribosome, cytoplasm, intracellular, neurogenesis, structural molecule activity, translation, cell differentiation, biological_process, molecular_function, structural constituent of ribosome, biosynthetic process, cellular_component, cytosolic large ribosomal subunit, anatomical structure development, cell, organelle	Ribosomal protein L30, ferredoxin-like fold domain, Ribosomal protein L7, eukaryotic

* Gene names were obtained from http://flybase.org.

** http://www.uniprot.org.

*** According to Gene Expression Atlas, http://www.ebi.ac.uk/gxa.

**Table 3:** Relative mRNA level for 8 genes under different influences.

Gene	Influence	Gender	R
*sug*	Ionizingradiation	M	2.4 (1.9–3.1)
		F	1.9 (1.7–2.1)
*ttk*		M	1.2 (1.0–1.4)
		F	2.0 (1.9–2.1)
*ana*	dioxin	M	2.2 (1.9–2.5)
		F	1.9 (1.7–2.1)
*CG16727*		M	2.6 (2.4–2.8)
		F	1.9 (1.6–2.2)
*Cyp12d1-p*	toluene	M	1.6 (1.5–1.7)
		F	1.6 (1.5–1.7)
*Fer3*		M	2.0 (1.9–2.2)
		F	1.5 (1.4–1.7)
*CG2065*		M	1.8 (1.6–2.1)
		F	1.5 (1.4–1.6)
*CG34447*		M	1.8 (1.7–1.9)
		F	2.4 (2.0–3.0)

Note: qPCR data. R – relative mRNA level (n-fold increase), in parenthesis a range of mRNA level differences is shown. M – male; F – female.

## Discussion

Our data revealed both significant similarities and differences in differential gene expression and the activity of biological processes under the influence of ionizing radiation, dioxin, formaldehyde, and toluene. The similarity of ionizing radiation, toluene, and formaldehyde may be because all three induce oxidative stress in cells and cause DNA damage. DNA is the most vulnerable structure of the cell. DNA determines cellular activity by coding proteins, but a cell has only two copies of each DNA molecule. Hence, while intact copies can replace other damaged macromolecules, DNA damage can lead to disruptive consequences. Genotoxicants cause heritable (across generations of cells or organisms) changes that affect human health and biodiversity of biota; they cause heritable adverse effects in offspring, the occurrence of cancer, and accelerated aging [39]–[41]. At the same time, due to DNA or other cell-structure damage, the cell stress response occurs, including sensing damage and the induction of the expression of certain genes. Adverse factors can damage intracellular signaling proteins that mediate the regulation of gene expression. For example, ionizing radiation is capable of causing the formation of reactive oxygen species that damage protein, including regulatory protein [42]. Formaldehyde promotes the formation of protein-protein and DNA-protein crosslinks [43], [44]. 2,3,7,8-tetrachlorodibenzo-p-dioxin, which is not directly genotoxic, binds to the aryl hydrocarbon receptor AhR, an intracellular protein that is a transcriptional enhancer affecting the expression of important genes [10], [45], [46].

Some of the observed transcriptional changes in stress can be regarded as protective and adaptive in nature (cell cycle arrest, induction of antioxidant and DNA repair systems, molecular chaperones), while the rest are related to the dysfunction of cellular systems (violation of redox and biosynthetic processes). In our previous *Drosophila* experiments we observed low doze radiation protective effect on lifespan [17], [47], [48]. We can consider the following as examples of protective changes identified in the present experiment: the changes in the levels of gene expression of proteasome, nucleotide and base excision repair, mismatch repair, ubiquitin-dependent proteolysis, heat shock proteins, and basal transcription factors.

As mentioned before, ascorbate and aldarate metabolism and retinol metabolic pathways are present in the dioxin, formaldehyde, radiation, and toluene gene lists. Previous studies demonstrated the correlation between all these types of stress and retinol metabolism [49]–[51], but there is no information about the correlation between these types of stress and the ascorbate and aldarate metabolic pathway. This leads us to believe that this is a new unknown marker of dioxin, formaldehyde, and toluene treatments. In living organisms, ascorbate acts as an antioxidant by protecting the body against oxidative stress [52]. Ascorbate also plays a role in oxidative protein folding in the endoplasmic reticulum [53].

We observed altered metabolism of xenobiotics by cytochrome P450 under the influence of dioxin and toluene in females and, to a lesser extent, in males. From previously published data, we know that 2,3,7,8-tetrachlorodibenzo-p-dioxin dose-dependently induces expression of cytochrome P450 1A1 [54].

The changes in some of the signaling pathway genes are most likely adaptive as well. We have seen changes in TOR-signaling and cell differentiation signaling (Notch, TGF-beta, Jak-STAT, Hedgehog, Wnt). For example, activation of Sonic hedgehog signaling protects carcinoma cells against ionizing radiation [55]. A change in TGF-beta signaling has been previously detected after radiation [56] and dioxin influences [57]. Changes in Notch signaling have been previously identified under the action of ionizing radiation [58]. Activation of the Jak/STAT pathway has been previously detected after exposure to high-density neutron irradiation [58]. The Wnt pathway has been activated by X-rays [59] and dioxin [54]. Despite previous work demonstrating alterations in the mentioned signaling pathways after various kinds of stress, the change in the four studied factors was detected first in this work. We can assume that adaptive changes are reproducible for different stressors, because they were formed as a result of the long evolution of a non-specific stress response.

The effects of cell malfunctions have a stochastic or a sex- and stress-specific character. For example, glycolysis and the metabolism of both tyrosine and phenylalanine changed primarily under the influence of dioxin and toluene. However, the change in the metabolism of aspartate, glutamate, and alanine was sex-specific (some genes went up, some down): in females after exposure to dioxin and toluene, and in males after exposure to irradiation. Dysfunction likely relates to the observed change in the gene expression of DNA replication, ribosome, RNA transport, and biosynthetic pathways (synthesis of various amino acids and nucleotides).

In the analysis of differentially expressed transcripts, we prepared a list of genes unique to each treatment that reproducibly increase or decrease their levels in both males and females (Table 2). For 8 of these genes expression level alterations were validated using qPCR (Table 3). In the case of radiation exposure, both sexes reproducibly exhibited increased expression of the transcription factors *sugarbabe* and *tramtrack*. Sugarbabe is a transcriptional repressor involved in the regulation of nutrient response and starvation-induced stress [60]. Tramtrack transcription factor controls Notch-dependent cell cycle regulation [61]. After irradiation decreased expression of the genes responsible for immunity (*CG18180, Cyp6a20*), lipid metabolism (*CG6295, CG6675)*, oxidative stress response (*CG9360, Keap1, Glutathione S transferase E3*), aggressive behavior (*Cyp6a20*), detoxification of synthetic insecticides (*Cytochrome P450-6a2, Glutathione S transferase E3*), inhibition of transcription and several genes with unknown function (Table 3). Under dioxin treatment, the metabolic gene *anachronism*, *CG16727*, and several genes with unknown function were up-regulated. Such genes as *CG12519, CG18048, Helix loop helix protein 3B* were down-regulated (Table 3). Toluene activated the toxic response gene *Cyp12d1-p*; transcription factor *Fer3*’s gene; the metabolic genes *CG2065*, *CG30427*, and *CG34447;* and the genes *Spn28Da* and *Spn3*, which are responsible for reproduction and immunity but deactivated genes responsible for formation of external skeleton (*Chitin deacetylase-like 9, Cuticular protein 72Ec*), phosphodiesterase activity (*CG15533, CG15534*), gustatory response (*Gustatory receptor 39a*), cholesterol metabolism (*Niemann-Pick type C-1b*), functioning of nervous system, (*CG10357*, *Ribosomal protein L7-like, Dopamine 2-like receptor*), nucleotide metabolic process (*CG34423*), vitelline membrane integrity (*Chorion protein b at 7F*), immune response (*Osiris 8*) (Table 3). It is interesting to note altered metabolism of cytochrome P450 under the influence of dioxin and toluene in females and, to a lesser extent, in males. From previously published data, we know that 2,3,7,8-tetrachlorodibenzo-p-dioxin dose-dependently induces expression of cytochrome P450 isoforms [62]. CYP450s use electrons from their cofactors to catalyze activation of molecular oxygen, leading to substrate oxidation for different goals - steroid hormones synthesis, degradation of xenobiotics (organic toxins).

In our opinion, list of genes unique to each treatment can be considered a gene expression signature and can be used for biomonitoring and biosensing of dioxins, toluene and low doses of radiation, in the model *Drosophila*.

## Supporting Information

Table S1 **Significant gene lists (with, FDRs and log fold changes).** *Notes:* muA, muB - mean of normalysed read numbers, lfc indicates the logarithmic foldness of UP- or DOWN-regulation, difExpr - the differences between comparison groups, stats - wald test statistic, pval – p-values. IM, DM, TM, FM, IF, DF, TF, FF are abbreviations of irradiation (I), dioxin (D), toluene (T) and formaldehyde (F) treatments for males (M) and females (F), accordingly. *Notes:* Genes involved in response more than 2 different influences are highlighted in bold. GO functions for gene lists are obtained from online tool g:Profiler (http://biit.cs.ut.ee/gprofiler/index.cgi) IM, DM, TM, FM, IF, DF, TF, FF are abbreviations of irradiation (I), dioxin (D), toluene (T) and formaldehyde (F) treatments for males (M) and females (F), accordingly.
(XLSX)

Table S2 **The list of overlapped genes for different influences and their functions.** *Notes:* Genes involved in response more than 2 different influences are highlighted in bold. GO functions for gene lists are obtained from online tool g:Profiler (http://biit.cs.ut.ee/gprofiler/index.cgi). IM, DM, TM, FM, IF, DF, TF, FF are abbreviations of irradiation (I), dioxin (D), toluene (T) and formaldehyde (F) treatments for males (M) and females (F), accordingly.
(DOCX)

Table S3 **List of KEGG pathways, corresponding to top100 differentially expressed genes after every treatment before enrichment procedure.** *Notes:* IM, DM, TM, FM, IF, DF, TF, FF are abbreviations of irradiation (I), dioxin (D), toluene (T) and formaldehyde (F) treatments for males (M) and females (F), accordingly.
(XLSX)
