# Supplementary material for: Mining Gene Expression Data for Pollutants (Dioxin, Toluene, Formaldehyde) and Low Dose of Gamma-Irradiation
Source: PLoS One. 2014 Jan 24;9(1):e86051. doi: 10.1371/journal.pone.0086051 (PMC3901678; doi:10.1371/journal.pone.0086051)
Supplement: Table S2 — The list of overlapped genes for different influences and their functions. Notes: Genes involved in response more than 2 different influences are highlighted in bold. GO functions for gene lists are obtained from online tool g:Profiler (http://biit.cs.ut.ee/gprofiler/index.cgi). IM, DM, TM, FM, IF, DF, TF, FF are abbreviations of irradiation (I), dioxin (D), toluene (T) and formaldehyde (F) treatments for males (M) and females (F), accordingly. (DOCX) [file pone.0086051.s002.docx]

Supplementary Table 1. The list of overlapped genes for different influences and their functions.

| Comparison | | Common genes | Gene Ontology Terms |
| --- | --- | --- | --- |
| IM_up | DM_up | **Hsp83,** ptr, **CG43343, mud, CG33017, Su(var)2-10, Msh6, oa2, blue, DnaJ-1, CG1924, CG32816,** CG14880, **ana1, del, Cul-3, CG15824,** CG10492, **ssp3,** Mes-4, **CG14215,** CG15548, **CG17150, CG15086, MCPH1,** ssh, prominin-like, CG41020, CG15376, CG32365, Dhc62B, CG17129, Dhc16F, CG15894, Pp2C1, Ubi-p63E, **lmg, Gr61a, CG13084,** CG8494, heph, Nek2, **nAcRbeta-64B, brp, CG34441, CG13995,** CG42232, Eps-15, **CG42402,** rib, **Lap1, CG42796** | Response to stimulus, regulation of transcription, serotonin receptor signaling, gamete generation, cell differentiation, protein ubiquitination, proteolysis, glycerol-3-phosphate catabolism, chitin metabolism, glucosamine metabolism, mitosis, microtubule-based movement, behavior |
| IM_up | TM_up | **Hsp83, CG43343, mud, CG33017, Su(var)2-10, Msh6,** Fs(2)Ket, **oa2, blue, DnaJ-1, CG1924, CG32816, ana1, del, Cul-3, ssp3,** Sox21b, **CG14215,** CG4022, CG30020, **CG17150, CG15086, MCPH1,** Su(var)2-HP2, CG33288, didum, **CG16778,** CG1632, apt, **lmg, Gr61a,** CG33125, CG8478, **CG13084,** CG14982, CG3288, scrt, **CG34371,** prd, **nAcRbeta-64B, brp, CG34441, CG13995,** Parg, **CG42402, Lap1, CG42796** | Response to abiotic stimulus, gravitaxis, mitotic cell cycle, microtubule-based movement, cell differentiation, Drosophila brain development, proteolysis, carbohydrate derivative catabolic process, glycerol-3-phosphate metabolic process, protein folding, gamete generation, JAK-STAT cascade, serotonin receptor signaling pathway |
| FM_up | DM_up | aret, **Imp** | Response to stimulus, synaptic growth, spermatogenesis |
| DM_up | TM_up | CG13792, **Hsp83, mud,** CG32681, CG14500, **oa2,** CG14669, mei-218, **lmg, CG15086,** Pkd2, **CG14215, blue, CG32816, CG42796, CG13330,** CG8516, mei-217, **del, Imp,** Plod, 5-HT1A, **CG34441, DnaJ-1,** CG10254, **nAcRbeta-64B, brp, CG1924,** CG6908, CG32686, CG5122, **CG17150,** Try29F, **CG42402,** CG5466, **CCHa1r,** CG30419, **beat-IIb,** CG33543, rdo, CG10384, **MCPH1, Cul-3, CG3078,** Tollo, CG16959, **Gr61a,** CG10864, **CG13995,** CG1600, **Lap1,** CG9906, **ssp3,** Cpr23B, **Su(var)2-10,** stg1, CR43087, CG4678, CG32036, **CG43343,** CG11566, CG31270, Dap160Msh6 endoB Hop CG12071 CG6621 CG31211 CG13084 CG8786 CG33143 CG31371 ana1 CG31760 yemalpha CheB93b CG6279 CG33017 CG34362 CG6752 CG43347 CG31817 CG11902 Nmdar1 Hsc70Cb CG15523 Hrs Obp99d robo3 | Reproduction and gamete generation, , response to stimulus, mitotic cell cycle, microtubule-based movement, cell adhesion, membrane invagination, cell differentiation, circadian rhythm, learning or memory, neurogenesis, oxidation-reduction process, chitin metabolism, proteolysis, glycerol-3-phosphate and GTF catabolism, G-protein coupled receptor signaling, RNA splicing, potassium ion transmembrane transport, phagocytosis, serotonin receptor signaling, regulation of protein stability |
| FM_up | TM_up | **Imp** | Synaptic growth, spermatogenesis |
| IF_up | DF_up | **Pepck, Npc2h, CG11459, Cyp4d21, CG11529, Ugt36Bc, malpha, alpha-Est2, CR40469, CG31664, CG11741,** Kr-h1, **CG15096, CG18302, Acp36DE, daw, Or85b, Spn42Db, GNBP3,** CR34335, **Gr39a, MtnC, Sodh-1, CG10581, CG18249,** snoRNA:Or-CD9b, **CG3523, CG15824, CG8509, ato, CG43317, CG7560, Glt, CG34331, CG32667, CG42305, CheB98a, CG32191, CG13455, yellow-f2, CG14205,** CG30047, N, **CG42868, CG3246, CG5527, CG7079, Pu,** snoRNA:Or-CD9a, **CG31326, loopin-1,** CG17781, **CG1773, mt:ATPase6, Cyt-b5-r,** CG9766, **snRNA:7SK, CG43114,** CG7906, CG12209, **CG14823, CG6415,** CG1673, **CG31183, abd-A, mir-2a-1, CG3213,** CG11714, **CG30431, CG30281, CG3348,** skpC, **Gr33a, Ela, CG34327,** Gr64a, **dar1,** Ir54a, CG8147, CG2652, **Ugt37a1, CG4415, Vago, CG9021,** CG1732, can, CG15219, **Obp83ef,** snoRNA:U14:30Eb, **CG8564, CG32833, grh, CG5707, CG43071, CG31778, CG30002, lectin-24A, CG43115,** CG13405, **Tequila,** CG12512, CG14627, **CG5390, CG12061,** CG7781, CG2120, mthl11, **CG13079,** CG42457, **CG30503, CG14629, B-H2,** CG14946, CG9389, Ahcy89E, **pug, CG3927, Spn88Eb, CG18682,** CG32088, CG1545, **CG42876, Rfabg, CG18536, CG13607, CG17325,** CG42561, ac, CG14644, **CG16836, CG3984, TfIIA-S-2,** CG13843, cpo, ninaD, **CG10252,** CG9780, **CG10680, CG13360, Rx,** CG10051, **CG14523, CG14400,** CG15673, **TotF, CG43123,** CG17917, **CG42816, HLH4C,** CG13077, **Dsx, fat-spondin,** ex, **TotA, CG14526, Ser7,** Gr10b, CG34025, **CG8745, Nplp2, CG14527,** CG6262, CG31007, **CG6333, CG42754, Cpr49Ae, CG10512, CG31414, CG43103, CR40679, Obp18a,** CG15042, **CG5171, CG34316, CG10924, His1:CG33801, CG2736,** ACC, **Fmo-1, Dll,** CG15544, CG9920, snoRNA:Me28S-A2113, CheA29a, **CR42653, pncr016:2R,** CG13035, **CG10962, CG2254, snoRNA:Or-CD11,** tw, **CG5973, CG7311, CG11659, dro2, CG9616, CG3597, CG6129,** snoRNA:Me18S-A28a, **CG17681,** Cwo, **Vha68-3, CG17244,** CG31954, scpr-C | Locomotion, response to biotic stimulus, response to chemical stimulus, response to stress, immune system process, protein localization, biological adhesion, cell communication, mitotic cell cycle, cell adhesion, programmed cell death, anatomical structure morphogenesis, oxidation-reduction process, carbohydrate metabolic process, lipid metabolic process, proteolysis, chitin metabolic process, glycerol-3-phosphate metabolic process, cyclic nucleotide metabolic process, protein folding, cellular amino acid catabolic process, transcription from RNA polymerase II promoter, learning or memory, gamete generation, regulation of JNK cascade, positive regulation of Notch signaling |
| IF_up | TF_up | **Pepck, Npc2h, CG11459,** CR32028, **Cyp4d21, CG11529, Ugt36Bc, malpha, alpha-Est2, CR40469, CG31664, CG11741, CG15096, CG18302, Acp36DE, daw,** d, **Or85b, Spn42Db,** gsb-n, **GNBP3, Gr39a, MtnC, Sodh-1, CG10581, CG18249, CG3523, CG15824, CG8509, ato, CG43317,** Acp53C14b, **CG7560,** CG5172, **Glt, CG34331, CG32667,** OdsH, **CG42305, CheB98a,** Spn77Bc, CG33648, **CG32191,** Abd-B, **CG13455, yellow-f2, CG14205, CG42868, CG3246,** yellow-e2, **CG5527, CG7079,** sc, **Pu, CG31326, loopin-1, CG1773, mt:ATPase6, Cyt-b5-r,** pnr, **snRNA:7SK, CG43114,** CG11475, ft, **CG14823, CG6415, CG31183,** CG4744, **abd-A, mir-2a-1, CG3213,** CG32591, **CG30431, CG30281, CG3348, Gr33a, Ela, CG34327, dar1,** Obp56f, CG42525, **CG4415, Vago, CG9021, Obp83ef,** e(y)2b, **CG8564, CG32833, grh,** CG31918, **CG5707,** CG30484, CG30486, **CG43071,** CG30280, **CG31778, CG30002, lectin-24A, CG43115,** CG43229, **Tequila, CG5390, CG12061,** CG13983, **CG13079,** CG34305, **CG34040, CG30503, CG14629, B-H2,** CG33342, CG43220, Gr64c, **pug, CG3927, Spn88Eb, CG18682,** CG43068, **CG42876, Rfabg, CG18536, CG13607, CG17325,** CG13296, CG42571, CG33958, **CG16836, CG3984, TfIIA-S-2,** Acp26Ab, CG31832, **CG10252, CG10680, CG13360, Rx,** CG10651, CG13245, **CG14523, CG14400,** CG13560, **CG11449, TotF, CG43123, CG42816, HLH4C,** CG14829, **dsx, fat-spondin, TotA, CG14526, Ser7,** CG31021, **CG8745, Nplp2, CG14527,** snoRNA:Psi18S-176, skpE, **CG6333,** CG31245, **CG42754,** vvl, Gr59f, **Cpr49Ae, CG10512,** Ir60a, **CG43103, CR40679, Obp18a,** Or82a, PebII, Gr47a, **CG5171, CG34316, His1:CG33801, CG2736, Fmo-1,** CG31235, **Dll,** CG5863, **CR42653, pncr016:2R, CG10962, CG2254,** CG5157, CG8219, CR40621, **snoRNA:Or-CD11, CG5973,** CG3565, **CG7311, CG11659, dro2,** elav, **CG9616, CG3597,** Gr39b, **CG6129,** CG34453, CG5262, **CG17681, Vha68-3, CG17244** | Response to biotic stimulus, response to chemical stimulus, response to stress, protein localization, cell communication, cell cycle, cell adhesion, cell differentiation, post-embryonic organ development, oxidation-reduction process, oligosaccharide metabolic process, chitin metabolic process, glycerol-3-phosphate metabolic process, cGMP metabolic process, cellular amino acid metabolic process, trehalose biosynthetic process, glucose metabolic process, proteolysis, chitin metabolic process, protein folding, transcription from RNA polymerase II promoterfatty acid biosynthetic process, learning or memory, organ growth, gamete generation, JNK cascade, Notch signaling pathway |
| DF_up | FF_up | CG18417 | Proteolysis |
| DF_up | TF_up | **Pepck, TotA,** TotC, CG17108, **Cyp4d21, Obp83ef,** Lsp1beta, **Npc2h, CG30503, Sodh-1, Men,** CG12256, **Ugt36Bc, CG32667,** CG5493, **CG5527, Cyp28d1, CG14629, fat-spondin,** CG10361, **CR40469,** Lip2, Yp3, **TotM,** Adhr, Adh, CG8550, nec, CG5327, **CG43114,** dro4, CG5428, Hsp70Bc, CG42846, **CG43071,** sPLA2, Galt, CG16762, CG9629, **CG10581,** Npc2g, **GNBP3, CG32191,** CG30083, **CG7079, CG13422,** CG32726, CG16772, CG13965, **CG15096, CG17681,** CG13461, **CG17244, CG3246, CG18302,** CG2145, Iris, **CG8745, pug,** CG6503, **CG2736,** CG4725, **Ela,** CG13308, **Ser7, Glt, CG14823,** Obp99c, CG14528, **Odc1, dar1,** CG9507, CG11594, **CG6415, CG34331,** CG7470, **CG3984, arg,** CG17032, CG5210, CG1544, CG3505, Hn, CG9914, CG31465, **CG6188, CG14400, CG1773, CG3523, CG42369, CG43103, CG11741, Tequila, CG3597, CG7560, CG31183, Rx, CG11659, Vago,** Lsp2, **CG18536,** CG31445, CG16978, Cyp313a1, **CG30431, Cyt-b5-r, CG31326, CG13877, Vha68-3,** CG2854, CG4723, **malpha,** dro5, CG43400, kappaB-Ras, snoRNA:Me28S-U2134b, CG11159, **Nplp2,** CG16756, Tret1-1, CG14439, fusl, **CG10680,** CG15202, **daw, CG16836,** CG15368, **CG14527,** CG9886, **alpha-Est2,** Obp44a, fit, Pfrx, CG13962, CG3999, **CG5840,** CG9928, CG10834, SIP3, **CG14526, CG6129,** btl, **CG3348,** CG42711, Faa, CG16704, IM3, Spat, noe, **Tps1,** Mtp, **Or85b,** CG14945, CG11236, CG18806, CG1648, **CG3239,** CG8586, P5CDh2, CG4000, **His1:CG33801, pyx,** CG32425, **CG3927,** CG43351, BM-40-SPARC, **yellow-f2,** CG31207, **TfIIA-S-2,** pinta, CG7135, **CG8129, CG14523,** CG3775, aralar1, **Fmo-1,** CG8358, desat2, **CG11529,** CG1468, CG16936, in, CG13101, grass, **dsx, Spn88Eb,** CG9505, IM4, **CG18249, CG11459, CG18682,** CG42467, Cyp6w1, **DnaJ-1,** Spn43Ab, Fkbp13, **lectin-24A,** CG30289, disco, Gel, CG43093, **CG13607,** CG15293, nimB2, **CG3213,** CG6435, snmRNA:763, CG5991, **CG34327,** TepII, CG9631, **Gr33a,** CG31150, CG5778, **CG43115,** Cyp309a2, **CG33307, CG14205, CG42305,** CG1461, **CG31778,** CG4927, **CG43317, CG5390,** CG3940, CG9691, Sr-CI, CG16743, CG42741, **Rfabg,** fon, CG32698, **CG4757,** Sgs4, **CG30002, CG15824,** emp, CG11395, Ance-4, Cyp6t1, CG6426, CG14259, CG33493, **CG31664,** CG2233, **Hf, ems,** CG8788, Kaz1-ORFB, CG15203, CG5321, Hex-C, CG34136, Cyp6g1, CG31705, **CG5945,** CG4716, **CG5707,** Pp1alpha-96A, LKR, CG31415, Gr93a, **CG42876,** CG12546, **CG10512,** CG31427, CG4019, CG7786, yellow-f, **pncr016:2R, CG12061,** CG33494, CG4250, CG13841, **CG17325,** CG18107, CG30324, Gpdh, antdh, Tsf1, **mt:ATPase6,** CG32521, psh, CG18067, **CG30281,** CG6495, CG12116, CG11842, Or92a, CR42873, **CheB98a,** nimC3, **CR42653,** AcCoAS, **Tsf3, CG9616, IM14,** CG15661, CG15067, PGRP-SB1, Obp99a, **HLH4C,** Hsp70Ab, **Pgm, CG42370, abd-A, CG5171,** CG1092, tobi, hgo, **CG34316, CG13360, snoRNA:Psi28S-2444,** moody, CG16926, CG10516, CG4847, CG4729, Galk, CG7016, CG14105, CR32194, CG43091, CG8738, CG42556, CG6429, GRHR, **CG13455, Dll,** Yp1, **Spn42Db,** CG15550, nAcRbeta-21C, nimB5, Pk1r, CG31777, CG31769, CG34376, CG12201, CG13794, desat1, Phlpp, CG11951, **CG2254,** CR43160, CG9372, **loopin-1,** UGP, CG18003, CG1791, **Pu,** Cyp4ac2, alpha-Est7, CG3513, **Obp18a, CG42868,** HDAC6, Pdfr, Idgf4, CG2574, CG3534, Hsp70Bbb, CG31313, ppl, CG10031, et, bgm, CG11841, CG12783, CG4721, CG11739, CG12693, **CG32833, CG13079,** CG34011, **CG6333, CG13641,** Tig, CG31693, Sox102F, NtR, **CG10962, CG8449,** CG14932, CG10799, **dro2,** skap, Ugt36Ba, CG5162, CG17928, **CG42816, CG32695,** yellow-e, SPE, CG4335, CR42875, Spn77Ba, snoRNA:Me28S-G1083c, CG17549, CG12140, Spn1, **Gr39a,** CG8654, ATPCL, CG31644, MtnB, MP1, CG6910, CG33932, Ir47a, CG5538, **CG8564,** eIF4E-3, CG14661, CG16712, retm, CG5565, **He,** CG42764, CG1516, **grh,** CG13085, Hsp70Bb, CG43062, CG12703, **Acp36DE, CG7311,** Tret1-2, CG4950, **snRNA:7SK, CG5973, CG15120,** Obp57b, CG6421, Ir20a, CG1139, **CG10252,** CG31741, CR41605, srp, Irc, CG10178, Cyp6a15Psi, CG33120, Sdc, CG4669, CG10700, **CR40679,** Eaat1, CG6115, **ato,** snoRNA:U29:54Eb, Lsd-1, **MtnC,** CG15917, CG2983, TrxT, CG12914, ap, CG4362, l(2)34Fc, **CG4415, snoRNA:Or-CD11, CG9021, TotF, CG43123,** CG5791, CG43255, CG9649, **CG42754,** snoRNA:Me28S-C3420b, CG13833, CG17646, mir-276a, CG30036, mt:CoIII, CG12643, CG31562, Fer2LCH, **CG10073,** CR43305, CG33928, **CG8509, CG30026,** CG12016, CG14567, Rpp20, **Cpr49Ae,** CG33137, sxe2, IM2, CG43092, CG32391, Sp212, spin, TTLL3B, **B-H2,** CG13912, **mir-2a-1,** CG31743, snoRNA:Me28S-C2645c, snRNA:U5:14B | Reproduction, immune system process, response to biotic stimulus, response to chemical stimulus, response to stress, protein localization, cell cycle, cell communication, cellular component movement, programmed cell death, Golgi organization, chromatin modification, extracellular matrix organization, tissue development, cell differentiation, amino sugar metabolic process, macromolecule modification, glucose metabolic process, macromolecule glycosylation, oligosaccharide metabolic process, protein maturation, proteolysis, protein modification process, gluconeogenesis, lipid biosynthetic process, chitin catabolic process, oxidoreduction coenzyme metabolic process, glycerol-3-phosphate metabolic process, acetyl-CoA metabolic process, GTP metabolic process, protein polyglycylation, protein phosphorylation, organic acid metabolic process, cellular amino acid metabolic process, cellular respiration, transcription from RNA polymerase II promoter, trehalose biosynthetic process, sensory perception of smell, multicellular organismal development, ion transport, gamete generation, Toll signaling pathway, |
| IM_down | DM_down | **CG6188,** Cyp4d14, **Damm, CG31809, CG31288,** Cyp6a8, **CG31104, CG9498, CG15199,** CG13658, **CG34236,** Jheh1, CG6733, **CG15043,** CG15422, **CG6839, Eip71CD, CG17224, CG13641,** PGRP-LB, **RpL5, CG5770, Cpr66D, Mal-B2,** CR43310, **CG14933,** GstE5, **CG14022, CG11192, CG11447, CG12766, CG15919, CG10827, CG10910, Hr38, CG42728, bmm, CG11878,** CG10560, **CG30272, maf-S, CG16985, GstD8, CG15282, CG4302,** Ak6, **CG15152,** CG5724, **Dpt,** CG11854, **CG11899, CG14630, kappaTry, Jon66Ci, CG1942,** m1, **l(1)G0230,** dnd, **CG6738,** CG7367, **CG34165, CG13587** | Response to biotic stimulus, response to stress, immune response, membrane invagination, tissue development, small molecule metabolic process, oxidation-reduction process, glycosyl compound metabolic process, lipid metabolic process, proteolysis, glycosaminoglycan catabolic process, RNA methylation, heterocycle metabolic process, organic acid metabolic process, endocytosis, regulation of transcription from RNA polymerase II promoter |
| IM_down | FM_down | **CG31810, CG31809,** CG2065, CG6145, CG4797, CG18404, **Eip71CD, Tsf3, Hr38, TotM, CG14630, CG8129,** CG4752 | Cell differentiation, nervous system development, small molecule metabolic process, oxidation-reduction process, steroid metabolic process, oxidoreduction coenzyme metabolic process, transmembrane transport |
| IM_down | TM_down | **CG6188, CG31810,** Obp99b, **Damm, arg, CG15120, CG31288, CG31104, CG32695, CG9498, CG15199,** CG11911, **Tps1, CG34236,** Ser6, CR14499, CG3106, **CG15043, CG6839,** CG42486, **Tsf3,** CG4259, CG18577, nimB3, CG34166, Ahcy13, **CG17224, CG14823,** CG30090, **RpL5,** CG10157, **CG5770, CG31414, Cpr66D,** RpL38, **Mal-B2,** CG6310, CG34026, RpL15, **CG14933, CG14022,** CG10824, Muc68E, CG33460, CG30285, CG43074, **He, CG11192,** CG14606, **CG11447, Odc1,** CG5189, **CG12766, CG15919, CG10827, CG10910, CG30026, CG42728,** CG34424, CG5921, pirk, Drs, **bmm, CG11878,** CG12224, salt, CG42649, **CG13422, CG30272, Pgm, maf-S, CG16985,** TwdlE, **GstD8, CG15282, CG4302,** CG34456, CecA2, sea, **CG15152,** Spn43Ad, fbp, CG11912, CG13102, **Dpt, CG11899, CG14630,** CG6048, **kappaTry,** CG43236, CG17664, Rel, **pyx,** UK114, **Jon66Ci, CG1942,** CREG, mRpS28, AttB, Nurf-38, CG42397, CG14245, **l(1)G0230, CG13877,** CG4653, RpL21, **CG6738,** CG4572, **Men,** CG32068, **CG34165, CG13587** | Response to chemical stimulus, response to amino acid stimulus, defense response to bacterium, RNA localization, membrane invagination, tissue development, amino sugar metabolic process, amino sugar metabolic process, lipid metabolic process, proteolysis, chitin metabolic process, RNA methylation, carboxylic acid metabolic process, cellular amino acid biosynthetic process, negative regulation of transcription from RNA polymerase II promoter, sphingolipid metabolic process, pyridoxine biosynthetic process, phagocytosis, dsRNA transport, regulation of gene expression, negative regulation of translation, steroid metabolic process |
| FM_down | DM_down | **CG11407, MtnD, CG14630, Eip71CD,** Obp56d, **CG31809,** CG32447, **Hr38** | Response to stimulus, oxidation-reduction process, steroid metabolic process, cell communication, G-protein coupled glutamate receptor signaling pathway, |
| DM_down | TM_down | CG42872, **MtnD, CG31288, CG15282,** GstD2, CG34172, CG2650, **CG9498,** CG32198, CG18538, **CG15043, CG6738, CG34236,** CG10659, **CG12766,** CG34239, **Cpr66D, CG11447,** CG9836, **CG42728, CG4461, Damm, CG15919, MtnC,** Hsp22, dob, CG30080, CG9172, CG3560, **CG6839,** CG14086, CG18636, **CG15199, CG1942,** CG43064, CG15044, CG17221, **CG14630, CG4302,** Ugt37b1, CG42867, CG31781, **CG6188,** PGRP-SB2, iotaTry, **CG14125,** CoVa, CG14401, CG15213, **GstD8,** CG7630, **CG30272, CG31104,** CG1946, **Mal-B2,** Spn31A, CG10664, CG9837, Oseg5, CG9203, Toll-4, CG5703, CG9396, CG15818, **CG34040, CG10073,** Cyp6d5, **Jon66Ci,** CG10163, CG14352, CG11340, **CG13587, CG10924,** CG9921, CG31300, CG33337, Ir85a, CG16732, CG5107, Npc2d, CG1550, **CG31326,** CG8206, CG32185, RabX5, CG34024, Or98a, **ems,** CG3817, CG42713, CG5018, CG3734, **CG8483, Ugt37a1,** CG33109, **CG33307, CG11529,** CR43364, CG33514, **CG11407,** CG14756, GstE4, CG15255, CG15545, spn-D, **CG14022,** CG17574, CG7920, CG16986, CG5910, CG6462, **CG11192,** CG12338, CG17726, CG34243, **CG42369, CG3239, CG7560, CG10910, CG8449,** mre11, CG31454, CG4169, CG7747, CG12780, **CG11878,** CR43242, CG43112, **CG17244,** CG15717, CG12164, CG32444, Cyp9h1, **CG14933, Cyp28d1, l(1)G0230,** pnt, **Dpt,** ZIP1, CG15023, l(1)G0255, **CG42370,** Vm26Ac, **CG5770,** CG42307, CG9903, **CG11449,** mt:ND6, Or24a, **maf-S,** ranshi, **CG15152,** CG9399, **CG16985,** mRpL13, l(2)35Di, **CG10827, CG34165, bmm,** Osi18, lush, Mes4, **CG11899,** Lcp65Ae, Hlc, **RpL5, kappaTry, Cpr49Ag,** CG34045, **snoRNA:Psi28S-2444,** CG12079, Mur11Da, **CG4757,** CG4364, CG30417, CG13041, CG1304, **Oat,** snoRNA:U27:54Eb, CG8317, **Hf, CG5840,** Tom, **CG5945,** l(2)06225, **CG17224, IM14** | Response to chemical stimulus, response to temperature stimulus, locomotory behavior, immune response, response to DNA damage stimulus, cell communication, cell adhesion, ribosome biogenesis, mitochondrial respiratory chain complex I biogenesis, zinc ion homeostasis, developmental process, circadian rhythm, nervous system development, oxidation-reduction process, amino sugar metabolic process, glycosyl compound metabolic process, glucose metabolic process, lipid metabolic process, proteolysis, protein modification process, gluconeogenesis, chitin metabolic process, phosphorylation, methylation, pyridoxine metabolic process, purine ribonucleotide metabolic process, GTP metabolic process, DNA repair, RNA splicing, carboxylic acid metabolic process, cellular amino acid metabolic process, acetyl-CoA metabolic process, regulation of transcription from RNA polymerase II promoter |
| FM_down | TM_down | **CG11407, MtnD, CG14630,** Jhe, CG13177, CG10472, Obp49a, CG6361, CG8834, **Tsf3, CG31810,** CG1140, CG14607, CG18537 | Oxidation-reduction process, glucosamine-containing compound metabolic process, proteolysis, steroid metabolic process, chitin metabolic process |
| IF_down | DF_down | **deltaTry,** CG32237, **Oat, CG42728,** CG14186, CG10405, CG13545, CG13227, CG11131, **Hr38, CG31809,** CG33270, CG5767, **CG14125,** CG33269, Muc55B, CG7715, **Ccp84Ab,** CG7298, GstD4, **CG31690,** GstD5, **CG11825,** CG4830, **CG13148, CG13202, CG14024, CG13026,** Hsp23, CG33271, CG9757, **CadN2,** CG6933, **CG17211, CG33483,** CG13311, **CG14947,** CG14394, CG32603, **CG34371,** CG14374, **Atf-2, CG15269,** CG42729, CG16798, Tango3, **CG15296,** CG43124, **sa,** CG34282, **CG12479, CG15484, CG4577, Pros28.1A,** CG8157, **CG13330, ref2, CG12419, CG15597, dpr11, CG10725,** CG6434, **CG11227,** CG14164, CG4375, **CG17816,** Map205, **dro6, Cha, Rgk3, Lsp1gamma,** CG13618, spok, **CG10694, Acp26Aa** | Response to stress, amino sugar metabolic process, proteolysis, steroid metabolic process, chitin metabolic process, JAK-STAT cascade |
| IF_down | FF_down | Npc2e | Peptidoglycan recognition protein signaling pathway, sterol transport |
| IF_down | TF_down | **deltaTry, Oat,** CG10621, **Hr38,** CG13325, CG13810, SA-2, CG8925, pwn, **Ccp84Ab,** CG1503, **CG31690,** CG43173, **CG11825,** CG5984, **CG13148, CG13202, CG14024,** CG18136, sage, **CG13026,** CG3280, CG12796, croc, **CadN2,** CG34432, trpgamma, CG3123, **CG17211, CG33483, CG14947,** CG10257, **CG34371, Atf-2, CG15269,** Cht2, CG12836, Rpt4R, CG1678, CG6191, Atg8b, **CG15296, sa,** CG12998, CG18179, CG30274, **CG12479, CG15484, CG4577, Pros28.1A,** CG13532, CG32547, CG33128, **CG13330, ref2,** CG13870, **CG12419,** CG13157, **CG15597,** snRNA:U2:14B, **dpr11, CG10725,** CG7953, CG13786, **CG11227,** CG12069, CG13999, CG13244, Sfp24Bb, **CG17816,** CG17140, **dro6,** CG7433, CG33173, Ir75c, CG8910, **Cha, Rgk3, Lsp1gamma,** CG15458, CG18109, CG32071, CG12484, CG12477, **CG10694,** CG33632, **Acp26Aa,** CG15537 | Response to stimulus, cell communication, cell cycle, negative regulation of apoptotic process, membrane invagination, microtubule cytoskeleton organization, regulation of hormone levels, cell morphogenesis, glucosamine-containing compound metabolic process, gene expression, proteolysis, carbohydrate metabolic process, chitin metabolic process, organic acid metabolic process, cellular amino acid metabolic process, phagocytosis, G-protein coupled receptor signaling pathway |
| DF_down | FF_down | **Nach, CG3323, Cad88C,** Vm32E | Sodium ion transport, calcium-dependent cell-cell adhesion, vitelline membrane formation involved in chorion-containing eggshell formation |
| DF_down | TF_down | **deltaTry, Nach, Oat, CG3323, CG13148,** CG42629, CG31262, Scp1, **Hr38,** CG1561, Act79B, Fmrf, Eh, **CG43347,** CG42749, **CG8483, Cad88C,** CG42286, Oli, CG9317, **CG13202,** hdm, **Atf-2,** CG11966, Oct-TyrR, tld, CG31068, **Cha,** Rab26, Hr46, **CadN2,** CG13830, Cpr47Ef, CG33082, PH4alphaNE1, CG5549, **CG4577,** CG12398, CG34347, CG7708, CG32709, Dscam2, **dpr11,** CG43223, ndl, trp, PIP82, CG32259, CG13862, CG6954, **CCHa1r,** nwk, **CG17211,** CG15323, Tehao, Ca-alpha1T, msta, **Ccp84Ab,** Gyc88E, **sa,** Dscam3, CG6441, GstD3, CG12858, CG34252, **CG13330,** wnd, stnB, Rh3, CG2993, CG15029, **Acp26Aa,** dys, CG13287, CG30127, CG18233, CG10362, **beat-IIb,** GstD6, **CG16778, oa2,** CG34357, CG8316, CG8177, stj, CG14047, **Lsp1gamma,** CG9813, **CG15484,** rab3-GEF, CG33465, mGluRA, Ror, CG9992, chp, CG33234, **CG14024,** CG10958, CG5953, CG2053, **Cpr49Ag,** CG41257, Elo68beta, Ilp7, Rim, CG9395, beat-Ic, **CG12479,** synaptogyrin, Tim17b1, snoRNA:Psi28S-2149, CG4641, **CG15269,** sens, **CG15597,** CG18139, Sclp, CG10184, Cpn, Ca-beta, **CG3078, CG31690,** beat-IIa, CG4596, sli, Pkcdelta, Aplip1, CG14054, **dro6, CG11227,** CR42549, CG14591, Eaat2, snoRNA:Psi28S-1837b, inaC, **Pros28.1A, CG33483,** CG14634, **CG17816,** Cpr49Ad, **Rgk3,** CG11052, CG33914, CG33203, hoe2, CG10654, CG11263, CG6123, **CG10725, ref2, CG10694,** CG15473, CG31115, **CG14947,** H2.0, CG13386, CG42640, CG5079, Lar, stnA, NnaD, CG34113, **CG15296,** CG5792, fas, **CG11825,** CG15546, CG42261, ocn, mmd, **CG34371, CG15919,** CG12682, CG15404, CG11426, CG2225, CG13721, CG31140, Pal2, CG18467, CG12119, **CG13026,** Pph13, kek6, CG34400, dec-1, Gbeta76C, CG17780, norpA, salr, CG12115, CG8087, **CG4461,** CG42377, CG11905, CG33791, Sfp78E, **CG12419,** Gas8, sns, Edg91 | Response to stimulus, response to heat, cellular response to light stimulus, cell communication, cell-cell signaling, membrane invagination, positive regulation of cell size, glucosamine-containing compound metabolic process, gene expression, acetate ester metabolic process, proteolysis, protein modification process, carbohydrate biosynthetic process, chitin metabolic process, dephosphorylation, phosphorylation, cGMP metabolic process, DNA and RNA metabolic processes, tricarboxylic acid cycle, acetylcholine biosynthetic process, gravitaxisamino acid transport, phagocytosis, negative regulation of gene expression |
| FF_down | TF_down | CG3906, **Cad88C, Nach, CG3323** | Sodium ion transport, calcium-dependent cell-cell adhesion |

*Notes:* Genes involved in response more than 2 different influences are highlighted in bold. GO functions for gene lists are obtained from online tool g:Profiler (<http://biit.cs.ut.ee/gprofiler/index.cgi>)

IM, DM, TM, FM, IF, DF, TF, FF are abbreviations of irradiation (I), dioxin (D), toluene (T) and formaldehyde (F) treatments for males (M) and females (F), accordingly.
